# Nose Bleed

**Published:** 1873-06

**Authors:** 


### NOSE BLEED, or Epsistaxis, as physicians love to call it, is an annoying symptom to many. The best possible treatment is to let it bleed, as it many times prevents an attack of apoplexy, and always, when spontaneous, is a relief to the system; nature, finding herself oppressed, thus endeavors instinctively to relieve herself of the injurious surplus. As long as the bleeding does not exceed half a tablespoon, make no effort to stanch the blood; if, however, it then continues, do not hold the head over a basin, as is universal, but hold it upright in a perpendicular position, and hold a cup under the nose, its rim touching the upper lip. Cold water may be snuffed partly up the nose and allowed to fall out again; a flat cold iron or piece of ice may be placed against the head, along the spine or between the shoulders, or place in the palm of the hand half a teaspoonful of the dust of a tea canister or caddy, unpulverized alum or finely powdered gum arabic, and snuff it up the nose. A large roll of paper pressed up against the nose under the upper lip, between it and the gums, is sometimes efficacious; or press the finger hard and persistently on the small artery at the wing of the nose on the bleeding side; or raise both the arms high above the head, and keep them in that position for some time; or soak a bit of lint or cotton in strong alum water, and plug up the nose; or apply a wet towel all along the spine, and renew as often as it gets warm. In slow and weakening diseases, a spontaneous nose bleed indicates great poverty of blood, and is a bad sign; but in all other conditions let the bleeding be followed by more exercise, half as much food, open air, and live entirely on fruits and coarse bread for a few days' at least; these aid in diminishing the excess of blood, and thus prevent imminent disease. In almost all cases, spontaneous nose bleed is a friendly warning of nature, and ought to be always heeded, and should be heeded as above indicated. First, let the bleeding continue for severel minutes. Second, secure a full, free evacuation of the bowels. Third, live on fruits and coarse breads for a few days.

